# Replication Capacity of Avian Influenza A(H9N2) Virus in Pet Birds and Mammals, Bangladesh

**DOI:** 10.3201/eid2112.151152

**Published:** 2015-12

**Authors:** Brian J. Lenny, Karthik Shanmuganatham, Stephanie Sonnberg, Mohammed M. Feeroz, S.M. Rabiul Alam, M. Kamrul Hasan, Lisa Jones-Engel, Pamela McKenzie, Scott Krauss, Robert G. Webster, Jeremy C. Jones

**Affiliations:** Rhodes College, Memphis, Tennessee, USA (B.J. Lenny);; St. Jude Children’s Research Hospital, Memphis (B.J. Lenny, K. Shanmuganatham, S. Sonnberg, P. McKenzie, S. Krauss, R.G. Webster, J.C. Jones);; Jahangirnagar University, Dhaka, Bangladesh (M.M. Feeroz, S.M.R. Alam, M.K. Hasan);; University of Washington, Seattle, Washington, USA (L. Jones-Engel)

**Keywords:** Influenza AH9N2, avian influenza, viruses, vector-borne infections, zoonoses, influenza, respiratory infections, mammals, pathogenesis, pet birds, poultry, mammals, surveillance, highly pathogenic avian influenza, H7N3, Bangladesh, India

## Abstract

Avian influenza A(H9N2) is an agricultural and public health threat. We characterized an H9N2 virus from a pet market in Bangladesh and demonstrated replication in samples from pet birds, swine tissues, human airway and ocular cells, and ferrets. Results implicated pet birds in the potential dissemination and zoonotic transmission of this virus.

Avian influenza A(H9N2) virus is endemic among poultry throughout Eurasia (*1*–*3*). In Bangladesh, subtype H9N2 viruses are unique reassortants, containing genes from highly pathogenic avian influenza A(H7N3) viruses. The H9N2 virus poses a substantial infection risk to poultry (*2*) and has infected pigs and humans (*4*,*5*). Its evolution is continually monitored by the World Health Organization (http://www.who.int/influenza/vaccines/virus/201502_zoonotic_vaccinevirusupdate.pdf?ua = 1).

Ongoing influenza surveillance in Bangladesh found H9N2 virus primarily in poultry (*5*,*6*); we also surveyed a pet market that sold avian pets (parrots, finches, pigeons) and poultry (quail, turkey, chickens) and obtained isolates from nonpoultry terrestrial birds (*6*). This mixture of birds and mammals, some for which little associated influenza pathogenesis data exists, provided a unique opportunity to study the ecology, host range, and transmission potential of H9N2 virus.

## The Study

We obtained H9N2 virus isolate A/environment/Bangladesh/9306/2010 (Env/9306) from a fecal sample collected from a parrot cage. Phylogenic data are available for other H9N2 viruses isolated in Bangladesh (*5*), but little phenotypic data exists for this lineage, which represents most H9N2 strains isolated in Bangladesh during 2010–2012. This strain clusters with isolates from Pakistan and India and has mammalian adaptations (*2*,*5*). We examined the pathogenicity of Env/9306 in birds commonly found at pet markets and assessed its capacity to replicate in and transmit among mammals by using ex vivo and in vivo models.

**Figure 1:**
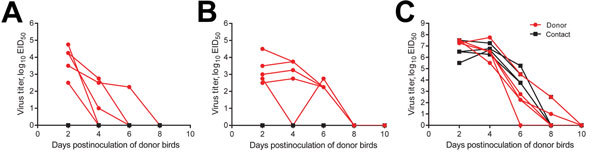
Oropharyngeal shedding of influenza A(H9N2) virus isolate A/environment/Bangladesh/9306/2010 (Env/9306) by pet birds and chickens, Bangladesh. Measurement of donor and contact bird virus shedding is based on the inoculation date of donors; donor and contact birds were kept in the same cage or enclosed environment. A) Donor finches (n = 5), B) parakeets (n = 5), or C) chickens (n = 6; red lines) were inoculated with 10^5^ log_10_50% egg infectious doses (EID_50_) units of Env/9306 and paired with naive birds of the same species (n = 4 or 5; black lines) in the same cage. Birds were swabbed every 2 dpi and virus titer (log_10_ EID_50_/mL) was determined in eggs. Individual shedding curves for each animal are provided.

To examine H9N2 replication in bird species, we inoculated 5 finches, 5 parakeets, and 6 chickens oculonasally with 10^5^ log_10_ 50% egg infectious doses (log_10_ EID_50_) of Env/9306 (Technical Appendix). Oropharyngeal and cloacal swab samples were collected every 2 days postinoculation (dpi) and titrated in eggs. Measurement of donor and contact animal virus shedding is based on the inoculation date of donors; donor and contact animals were kept in the same cage. Inoculated pet birds shed virus oropharyngeally (Figure 1) for 6 days, but not cloacally (data not shown). Chickens, a control H9N2 virus host, shed 2–3 logs more than did pet birds, and for a significantly longer time by area under the curve analysis (up to 10 dpi; p<0.001). Finches remained asymptomatic; parakeets and chickens showed sporadic clinical signs (lethargy, hunched posture, labored breathing) at 5–10 dpi. No birds died. 

Tissue samples were collected at 3 dpi (Table 1). Virus was isolated from the respiratory tract of 1 parakeet, 2 finches, and all 3 chickens, 2 of which had virus in the gastrointestinal tract. Virus was also isolated from the brain (2 finches, 1 chicken) and eye (1 finch, 1 chicken) (Table 1).

**Table 1:** Replication of avian influenza A(H9N2) virus in organs and seroconversion of inoculated birds, Bangladesh*

Bird	Organ titer†							HI titer‡	
	Brain	Eye	Trachea	Lung	Small intestine	Large intestine		Donor	Contact
Finch	2.9 (2/3)	3.5 (1/3)	3 (2/3)	3.5 (1/3)	–	–		4.3 (1/5)	– (0/5)
Parakeet	ND	ND	3.5 (1/3)	­–	–	–		6.1 (4/5)	5.3 (1/5)
Chicken	5.5 (1/1)	4.25 (1/1)	4.5 (3/3)	5.1 (3/3)	3 (2/3)	4.5 (1/3)		10.8 (6/6)	10.9 (4/4)

*HI, hemagglutination inhibition; –, below the limit of detection (<0.75 50% egg infectious doses [EID_50_]/mL or serum dilution <1:20); ND, not determined. †Tissues were harvested 3 days postinoculation and titrated in embryonated chicken eggs, and were reported as log_10_ EID_50_/mL. Data are the means of positive samples (>0.75 EID_50_/mL) (no. birds shedding/total birds sampled at given time point). ‡Mean reciprocal values (log_2_/50μL) of the highest titer that inhibited 4 hemagglutinating units of homologous virus (no. seropositive animals/total no. sampled).

Naive contacts of inoculated pet birds were not infected, but naive chicken contacts became infected and shed virus as early as 2 dpi (Figure 1). All birds were tested for seroconversion at 16 dpi by hemagglutination inhibition (HI) assay (*7*; Technical Appendix). Among finches, 1 of 5 donors and no contacts seroconverted. Among parakeets, 4 of 5 donors and 1 of 5 contacts seroconverted. All chickens seroconverted, and titers exceeded those of pet birds (Table 1).

To determine environmental shedding, we collected swab samples of drinking water, feces, and cages on 1–6 dpi. Virus was detected in water for finches (4 time points) and parakeets (1 time point) but not in fecal or cage swab samples, consistent with oropharyngeal shedding patterns (*8*). All chicken environmental samples contained virus for 4 of 6 time points (Table 2).

**Table 2:** Detection of avian influenza A(H9N2) virus in swab samples from environment of inoculated birds, Bangladesh*

Bird	Sample	Swab titer†					
		1 dpi	2 dpi	3 dpi	4 dpi	5 dpi	6 dpi
Finch	Water	3.25	2.5	2.5	1	–	–
	Cage	–	–	–	–	–	–
	Feces	–	–	–	–	–	–
Parakeet	Water	–	–	–	–	1	–
	Cage	–	–	–	–	–	–
	Feces	–	–	–	–	–	–
Chicken	Water	3.25	2.25	4.5	3.5	5.5	–
	Cage	3.5	4.5	4.5	4.5	3.25	2.5
	Feces	3	<	3.25	3.75	2.5	–

*dpi, days post-inoculation; –, below the limit of detection (<0.75 50% egg infectious doses [EID_50_]/mL). †Samples were titrated in embryonated chicken eggs and reported as log_10_ EID_50_/mL.

**Figure 2:**
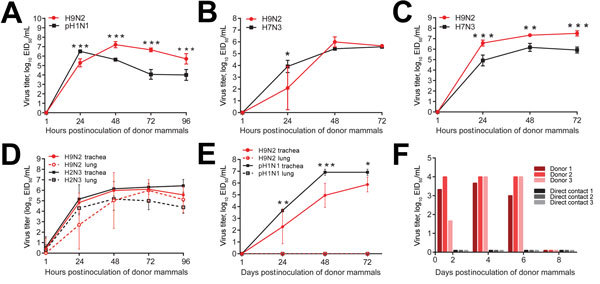
Pathogenesis of influenza A(H9N2) virus isolate A/environment/Bangladesh/9306/2010 (Env/9306) in ex vivo and in vivo mammalian models, Bangladesh. Replication kinetics of Env/9306 or a virus control are shown in A) primary normal human bronchial epithelial cells, B) primary human corneal epithelial cells, C) primary human trabecular meshwork cells, D) swine respiratory tissue explants, and E) ferret respiratory tissue explants. Error bars indicate mean + SD of the combined results of 2 individual experiments of n = 3 inserts, wells, or tissue explants per virus group. Env/9036 replication is indicated in red, and control virus replication in black. F) Replication of Env/9306 in ferrets (n = 3; red bars) and transmission to naïve, direct contact ferrets (n = 3; black bars) housed in the same cage. Statistical significance of replication between virus groups at a given time point was determined by performing a 2-way analysis of variance. *p≤0.05; **p≤0.01; ***p≤0.0001. EID_50_, 50% egg infectious doses.

The H9N2 virus strain Env/9306 contains mammalian-like mutations in genes, including HAQ226L (H3 numbering) (*5*), which increase H9N2 virus transmissibility to and among mammals (*9*). We modeled replication in humans (respiratory and ocular routes) by inoculating differentiated normal human bronchial epithelial cells (NHBEs) or primary human ocular cells (cornea and trabecular meshwork) with Env/9306 (multiplicity of infection 0.01) (Technical Appendix). The Env/9306 strain replicated in NHBEs to >7 log_10_ 50% tissue culture infectious doses (TCID_50_) per mL and exceeded titers of control human pandemic virus A/California/04/2009 (pH1N1) beyond 48 hours postinoculation (p<0.0001; Figure 2, panel A). Despite the higher titers, Env/9306 did not induce noticeable cytopatholgy in NHBEs, but pH1N1 did. In corneal and trabecular meshwork cells, Env/9306 replicated to similar titers as did control virus H7N2, a subtype previously shown to replicate in ocular cells (Figure 2, panels B, C) (*10*).

To assess replication in swine, we inoculated tracheal or lung tissue explants (Technical Appendix) from 1–2 week old piglets, with 10^5^ EID_50_/explant. Virus replicated >6 log_10_ TCID_50_/mL, comparable to a control swine H2N3 virus (Figure 2, panel D).

We modeled replication and transmission in vivo by inoculating 3 donor ferrets with strain Env/9306 (10^6^ EID_50_ units); each was co-housed with a separate naive, direct contact. Donors shed 4 log_10_ TCID_50_/mL in nasal washes for 6 dpi; 2 of 3 donors displayed lethargy, swollen sinuses, sneezing, or a combination of these during this period. No virus was shed by naïve direct contacts. One donor ferret displayed lethargy (4–8 dpi) and 1 sneezing (10–12 dpi) (Figure 2, panel F, data not shown). To examine whether the lack of transmission correlated with virus tropism, ferret tracheal and lung explants (Technical Appendix) were inoculated with 10^5^ EID_50_/explant of Env/9306 or pH1N1. Env/9306 replicated in ferret tracheal explants to titers >5 log_10_ EID_50_/mL (72 hours postinoculation), statistically lower than the rate for pH1N1 (Figure 2, panel E). No replication of either virus was observed in lung explants.

## Conclusions

We demonstrated replication of a nonpoultry avian influenza A(H9N2) virus in finches and parakeets with limited environmental shedding (water), but no transmission to cage mates. Shedding routes were more limited, virus titers lower, and clinical signs less frequent in pet birds than in chickens. Nevertheless, the potential for pet birds to act as vectors of the virus should not be underestimated. We recently showed that novel influenza A(H7N9) virus transmits between passerines, which include finches, and poultry by water despite a lack of intraspecies transmission (*8*); H9N2 virus has also been isolated from wild, finch-like birds in China (*11*).

Interspecies transmission of the Env/9306 strain remains a risk to mammals because of adaptation mutations (*5*,*9*) and is supported in this study by replication in ferrets and in human and swine tissues. Physical contact between pet birds and their owners, as well as shedding of virus into the environment (water), could be transmission sources.

Live bird markets are crucial to zoonotic spread of avian influenza viruses (AIVs) (*12*). However, our data suggest transmission potential in pet markets and vendor sites other than poultry markets; these sites may house birds infected with AIVs and should be included in future surveillance. Our results may also inform surveillance sample collection. Oropharyngeal samples were collected from pet birds; collecting environmental swabs alone may yield lower isolation rates or fail to detect this virus. H9N2 virus replication in pet birds also has implications for viral spread. Poultry are a major source of dissemination, but our data show domesticated or pet birds can harbor H9N2. Pet trading can extend across international borders and greatly expand the range of AIVs, as when H9N2 virus was repeatedly imported into Japan in infected parakeets (*13*). Finally, the unique influenza varieties among pet birds may provide more opportunities for H9N2 virus to gain novel genetic elements; this subtype has had remarkable levels of reassortment activity with influenza A(H7N9) and highly pathogenic avian influenza A(H5N1) viruses (*14*,*15*).

H9N2 virus will remain a threat in the foreseeable future. Efforts are needed to identify its presence in poultry and nonpoultry avian species. Phenotypic properties of these viruses, including replication ex vivo and in vivo, are a valuable supplement to existing genotypic data and further inform the risk for spread within avian and human populations.

Technical Appendix Methods for influenza-specific laboratory procedures; primary cell and tissue culture; inoculation and exposure of donor and contact animals; and statistical methods to determine the replication capacity of avian influenza A (H9N2) virus in pet birds and mammals in Bangladesh.
