# Supplementary material for: Replication Capacity of Avian Influenza A(H9N2) Virus in Pet Birds and Mammals, Bangladesh
Source: Emerg Infect Dis. 2015 Dec;21(12):2174–7. doi: 10.3201/eid2112.151152 (PMC4672412; doi:10.3201/eid2112.151152)
Supplement: Technical Appendix — Methods for influenza-specific laboratory procedures; primary cell and tissue culture; inoculation and exposure of donor and contact animals; and statistical methods to determine the replication capacity of avian influenza A (H9N2) virus in pet birds and mammals in Bangladesh. [file 15-1152-Techapp-s1.pdf]

Article DOI: http://dx.doi.org/10.3201/eid2112.151152

Replication Capacity of Influenza A(H9N2) Virus in Pet Birds and Mammals, Bangladesh

Technical Appendix

All animal experiments were approved by the St. Jude Children's Research Hospital Hospital Animal Care and Use Committee (Memphis, TN, USA) and performed in compliance with the policies of the National Institutes of Health and the Animal Welfare Act.

Viruses

All viruses were propagated and titrated in 10-day-old, embryonated chicken eggs, with 50% egg infectious doses (EID_{50}) determined by the method of Reed and Muench (Am. J. of Hygiene, v27, 1934, pp.493–497). A/Environment/Bangladesh/9306/2010 (parrot, H9N2) was subjected to 2 rounds of treatment with Newcastle disease virus (NDV) hyper-immune sera and a final seed stock prepared from a limiting dilution series in eggs. RNA was isolated from the final virus stock preparation and subjected to reverse transcription (RT)PCR (Qiagen, Valencia, CA) by using 2 pairs of NDV gene specific primers as described (J. of Vir. Meth. 86(1), 2000, pp.71–83) to confirm the absence of NDV. Several control viruses were used to demonstrate productive replication in NHBE and ferret explants (A/California/04/2009, pandemic H1N1), swine explants (A/swine/Missouri/2124514/2006, H2N3), or ocular cells (A/LaughingGull/DE/42/2006, H7N3).

Cell Lines

Madin-Darby canine kidney (MDCK) cells (ATCC, Manassas, VA) were cultured in modified Eagle's medium (MEM; CellGro, Herndon, VA) supplemented with 5% fetal bovine serum (FBS; Thermo Scientific HyClone, Logan, UT) at 37°C in 5% CO_2. Virus titers from NHBE, ocular cell, explant, and ferret nasal wash infections were titered in MDCKs in MEM supplemented with 1% bovine serum albumin (BSA) and 1 μg/mL tosyl phenylalanyl

chloromethyl ketone (TPCK) treated trypsin. Titers in MDCKs were expressed as 50% tissue culture infectious doses ($TCID_{50}$) as calculated by the method of Reed and Muench.

Primary normal human bronchial epithelial (NHBE) cells (Lonza, Walkersville, MD) from a 4-year-old healthy male donor were cultured in an air-liquid interface (ALI) system on Transwell inserts (Corning, NY). The apical surfaces were exposed to a humidified 95% air and 5% CO_2 environment for a minimum of 6 weeks. Basal surfaces were cultured in BEBM medium (Lonza) supplemented with SingleQuot growth factors (Lonza). Basal medium was changed every 48 h along with an apical surface wash to remove mucus.

Primary human corneal epithelial and trabecular meshwork cells (Science Cell, Carlsbad, CA) were cultured in manufacturer supplied media supplemented with FBS at 37°C in 5% CO_2. During virus infections, cells were switched to serum free-medium supplemented with 1% bovine serum albumin and 0.5 μg/mL TPCK treated trypsin.

Explant Preparation

Piglets (1–2 week old, kindly provided by C. Leffler and E. Parfenova [University of Tennessee, Memphis, TN]) and ferrets (6–8 months old [Triple F Farms, Sayre, PA) and were euthanized. The trachea and lung lobes were removed intact and washed with PBS containing 10× penicillin-streptomycin-amphotericin B (Sigma, St. Louis, MO). The tracheas were opened by a single lengthwise incision, and explants (5 mm diameter) were prepared with a biopsy punch. One lower lung lobe was inflated and washed through an incision in the branching bronchus with PBS containing 10× antibitoics/antimycotic and then inflated again with 1.6% low-melt agarose (Sigma). After the agarose had solidified, 2×1×1 cm segments were cut from lung and embedded in a 4% agarose plug. Thin slices were cut from the agarose/lung plug and explants (5 mm diameter) were prepared from these slices. Tracheal and lung explants were maintained on transwell inserts, and 1 mL of medium (as described for NHBE culture) was added to the basal chamber. Tissues were incubated at 37°C in 5% CO2 for at least 18 h before infection.

NHBE and Explant Infection

The apical surfaces of NHBEs or explants were washed with PBS and equilibrated at 37°C for 30 min with infection medium (BEBM supplemented with 0.5% BSA). Cells and tissues were inoculated with 100 μL of virus inoculum at MOI 0.01 (NHBEs) or containing 10^5 EID_{50} units (explants) for 1 hr. Apical surfaces were given 2 acid washes (PBS, pH 2.2) and 3 pH neutral PBS washes to remove unbound virus. At each time point, 300 μL of infection was added to the apical surface for 30 min and harvested for titration in MDCKs. NHBE cell data are representative of combined data from 2 independent tests of 3 inserts per virus group. Explant data are representative of 2 independent tests with 3 explants per tissue per virus group.

Hemagglutination Inhibition Assay

The presence of anti-influenza hemagglutination protein antibody titers were determined by preparing serial 2-fold dilutions of receptor destroying enzyme (Denko Seiken, Campbell, CA) animal sera in PBS in ‘v-bottom’ shaped plates. Virus was standardized to 4 hemagglutinating units by using horse erythrocytes (Rockland, Limerick, PA) and added to the sera dilutions for 30 minutes. Horse erythrocytes were then added and incubated for 60 minutes. HI values were recorded as the reciprocal of the highest dilution of sera that inhibit virus-induced erythrocyte agglutination.

Avian Species Infection

Mixed age and sex zebra finches (n = 5, *Taeniopygia guttata*) and parakeets (n = 5, *Melopsittacus undulates*) (Birds Express, South El Monte, CA), 8–10 week old mixed sex white leghorn chickens (n = 5, *Gallus gallus domesticus*) (McMurray Hatchery, Webster City, IA) were quarantined for at least 1 week before experiments. Swabs from each bird were verified influenza negative by egg isolation. Food and water was provided ad libitum. Birds were inoculated intranasally, intraocularly, and orally with 10^5 log_{10} EID_{50} units of virus in 100 μL (finches, parakeets) or 500 μL (chickens) of phosphate buffered saline. All bird species were housed with a naïve contact animals (n = 4–5) to assess transmission. Oropharyngeal and cloacal swab samples were collected on days post inoculation (dpi) 2, 4, 6, 8, 10, and 13. Water samples

(500 μL), feces and cage swabs were obtained 1–6 dpi from each species' housing. Samples were titrated in eggs.

Ferret Infection

Male ferrets, 6–8 months of age (Triple F Farms), were verified seronegative for influenza A (H1 and H3 subtypes) by HI assay. Three donor ferrets in individual cages were lightly anesthetized by isoflurane and intranasally inoculated with 10^6 EID_{50} units of virus in a total volume of 500 μL. Twenty-four hours later, a naïve cage mate (direct contact) was introduced into the cage of each donor ferret. Every 48 hr, the ferrets' temperatures and weights were recorded before they were sedated with ketamine (25 mg/kg of bodyweight) and induced to sneeze by intranasal instillation of PBS. Virus titer in the nasal washes were titrated in MDCK cells.

Statistics

Mean infectious titers were compared by using the 1-tailed Student t-test in Excel (Microsoft, Redmond, WA, USA) or 2-way ANOVA GraphPad Prism v6 (La Jolla, CA, USA). Area under the curve analysis for cumulative shedding was performed by using GraphPad Prism v6.
